# The *Sirt6* gene: Does it play a role in tooth development?

**DOI:** 10.1371/journal.pone.0174255

**Published:** 2017-03-29

**Authors:** Xueyang Liao, Bo Feng, Demao Zhang, Peng Liu, Xuedong Zhou, Ruimin Li, Ling Ye

**Affiliations:** 1 State Key Laboratory of Oral Diseases, National Clinical Research Center for Oral Diseases, West China Hospital of Stomatology, Sichuan University, Chengdu, China; 2 Department of Endodontics, Stomatology Hospital, General Hospital of NingXia Medical University, Yinchuan, China; Università degli Studi della Campania "Luigi Vanvitelli", ITALY

## Abstract

Dental Mesenchymal Cells (DMCs) are known to play a role in tooth development as well as in the repair and regeneration of dental tissue. A large number of signaling molecules regulate the proliferation and differentiation of DMC, though the underlying mechanisms are still not fully understood. Sirtuin-6 (SIRT6), a key regulator of aging, can exert an impact on embryonic stem cell (ESC) differentiation. The experimental deletion of *Sirt6* in mouse bone marrow cells has been found to have an inhibiting impact on the bone mineral density and the osteogenic differentiation of these cells. The possible role of *Sirt6* in tooth development, however, has at present remained largely unexplored. In the present study, we found that SIRT6 had no effect on tooth development before birth. However, *Sirt6* gene deletion in knockout mice did have two post-natal impacts: a delay in tooth eruption and sluggishness in the development of dental roots. We propose an explanation of the possible molecular basis of the changes observed in *Sirt6*^-/-^ mice. SIRT6 is expressed in mouse odontoblasts. *Sirt6* deletion enhanced the proliferation of DMCs, as well as their capacity for adipogenic differentiation. On the other hand, it inhibited their capacity for *in vitro* osteogenic/chondrogenic differentiation. Further studies suggested that other factors may mediate the role of *Sirt6* in odontogenesis. These include the nuclear factor kappa B (NF-κB), p38 mitogen-activated protein kinase (p38-MAPK), extracellular regulated MAP kinase (ERK) pathways and the mitochondrial energy. We demonstrated that *Sirt6* plays a role in tooth root formation and confirmed that SIRT6 is necessary for DMC differentiation as well as for the development of the tooth root and for eventual tooth eruption. These results establish a new link between SIRT6 and tooth development.

## Introduction

Tooth development is a long, complex biological process that includes epithelial and mesenchymal interactions, cell differentiation, morphogenesis, tissue mineralization, and tooth eruption. Throughout all stages of this process, a series of complex biochemical reactions occur within the cell, which trigger physiological processes and play a crucial role in tooth development. Many signaling pathways and molecules are now known to play important roles in this process [1–3]. Numerous studies have reported the roles of Dental Mesenchymal Cells (DMCs) in tooth development. Several types of DMCs have already been identified from different sources. Among these sources are dental pulp, periodontal ligament, exfoliated deciduous teeth, dental follicle (DF), root apical papilla, human periapical cysts and dental bud [4–11]. Similar to embryonic stem cells, the proliferation and differentiation of DMCs is associated with biological molecules. The interplay between DMCs and these molecules remains largely unknown.

SIRT6 is a member of the sirtuin (SIRT) family of nicotinamide adenine dinucleotide (NAD+)-dependent deacetylases. It not only plays a role in maintaining the stability of genome and telomere function, in controlling the metabolism of glucose and lipid, and in regulating inflammation. It also suppresses the development of tumors and regulates the lifespan. Mostoslavsky et al (2006) were the first to suggest that sirtuin regulates aging in mammals. *Sirt6* knockout mice at birth are small in size and show no abnormalities. At 2–3 weeks of age, however, degenerative changes begin to emerge, including profound lymphopenia, loss of subcutaneous fat, osteoporosis associated with a 30% loss of bone mineral density, lordokyphosis, and severe metabolic defects. At approximately 4 weeks of age, the SIRT6 knockout mice die [12]. Additional studies in mice have indicated that SIRT6 deficiency affects the liver and nerves, leading to abnormalities in lipid metabolism, to adiposis hepatica, and to growth retardation [13, 14]. SIRT6 directly regulates the expression of several core pluripotent genes, including organic cation/carnitine transporter4 (Oct4), sex determining region Y-box 2 (Sox2) and Nanog. It does this via acetylation of histone H3 lysine 56 (H3K56ac), which controls ESC differentiation through ten-eleven translocation enzymes (TETs) -mediated oxidation of 5-methylcytosine (5mC) into 5-hydroxymethylcytosine (5hmC) [15].

DMCs not only have the ability to differentiate into odontoblasts but also share the same surface markers and characteristics with bone marrow mesenchymal stem cells (MSCs) [16, 17]; it can also interact with epithelial cells to affect tooth development. Despite the remarkable effects of SIRT6 on MSCs such as the ESC, the role of SIRT6 in DMCs and tooth development still remains unclear and merits further study. In this study, we have focused on elucidating the role of the SIRT6 protein on tooth development.

## Materials and methods

### Animals

SIRT6 global knockout mice (129/SvJ) were purchased from the Jackson Laboratory. The preparation and genotyping of mice were carried out in accordance with procedures described in previous reports [12]. *Sirt6* homozygous knockout (KO; *Sirt6*^-/-^) and wild-type (WT; *Sirt6*^+/+^) mice were prepared by heterozygote breeding. Mice were allowed access to water *ad libitum* and were fed a pathogen-free rodent diet. All animal experiments were performed according to approved protocols by the National Key Laboratory of Biotherapy, Core Facility of Gene Engineered Mouse (Chengdu, CN).

### Tissue preparation and histology analysis

For the samples of histology and immunohistochemistry, the mandibles or heads of embryonic, new born, and postnatal mice (both of WT and KO mice) were fixed overnight in 4% buffered paraformaldehyde. Samples of age 7 days postnatal (dpn) or older were decalcified in a buffered 10% EDTA solution from 5 days to 3 weeks. Tissues were dehydrated using graded ethanol, embedded in paraffin, serially sectioned, and stained with hematoxylin eosin. For immunohistochemistry, paraffin sections were dewaxed in xylene, rehydrated with distilled water, and then subjected to antigen retrieval for 30 min at 95°C. The slides were subsequently incubated overnight at 4°C with the anti-SIRT6 (1:1000dilution, Novus Biologicals, USA). Slides were then treated with an anti-rabbit secondary antibody (1:1000 dilution, Cell Signaling Technology Inc, MA, USA) and were developed using avidin-conjugated horseradish peroxidase **(**HRP) with diaminobenzidine (DAB) as a substrate (ZSGB-Biology, Beijing, China). This was followed by hematoxylin counterstaining.

### Micro-Computed Tomography analysis

Three-week-old male WT and KO mice (n = 8) were sacrificed by decapitation after etherization and the jaws were promptly fixed in 4% formaldehyde. The local ethical committee at Sichuan University approved all laboratory animal procedures. The histological observational area was imaged by using a Desktop cone-beam Micro-Computed Tomography System (VIVA-μCT40, SANCO Medical AG, Brüttisellen, Switzerland) with a scanning resolution of 10μm. The mesial and distal roots of the mandibular first molar were manually located at 20mm intervals on individual images. The selected images were compiled into a three-dimensional image. The data were analyzed with software (Materialise Mimics 13).

### Culture of primary DMCs

DMCs were isolated from the dental germs of 3-day-old WT and KO mice. Before surgery, the mice were sacrificed by cervical dislocation after anesthesia and sterilized using 75% ethanol for 5 min. Dental mesenchymal tissue was separated from the mandibular molar tooth germ by mechanical isolation, followed by digestion with type I collagenase (2mg/ml) (Sigma-Aldrich, MO, USA) for approximately 45min as previously described [18]. The tissue pieces were seeded into a 60mm cell culture dish (Corning, Tewksbury MA, USA), and cultured in Dulbecco’s modified Eagle’s medium (DMEM, Thermo Scientific, Waltham, USA), supplemented with 15% fetal calf serum (FCS, Gibco, Life Technologies, Carlsbad, CA, USA) and antibiotics (100 U/mL penicillin and 100 mg/mL streptomycin), grown in a humidified atmosphere at 37°C with 5% CO_2_. Experiments were carried out using DMCs between the third and fifth passages.

### Cell proliferation by CCK8 assay

Two groups of DMCs were cultured with a growth culture medium in 96-well plates (Corning, Tewksbury MA, USA) at an initial density of 3×10^3^ cells per well. Cell proliferation was evaluated using the cell counting kit-8 (CCK-8, Dojindo, Kumamoto, Japan) following the manufacturer’s instructions. The absorbance of A450 was measured using a microplate reader (Bio-Tek instrument, Winooski, USA) at various points in time (1–5 days).

### Flow cytometry

Cell cycle and apoptosis were quantified by flow cytometry. Two groups of DMCs were incubated with trypsin-EDTA. Cells were stained with propidium iodide (PI) or Annexin V-PE and 7-Amino-Actinomycin D (KGA511, KGA1015; KeyGEN Bio-TECH, Nanjing, China) following the manufacturer’s instructions. Flow cytometry analysis was performed on more than 50,000 events. The data were analyzed with system software (Millipore Guava Casyctye HT, Merck Millipore, Darmstadt, Germany).

### EdU incorporation assay

Two groups of DMCs were cultured in 6-well plates (Corning, Tewksbury MA, USA) at an initial density of 1×10^5^ cells per well with a growth culture medium after 3–5 days, 50μM 5-Ethynyl-2’-deoxyuridine (EdU) which were added from the Cell-Light^™^ EdU Kit (Ribo BIO, Guangzhou, China) and incubated for 2 hours. After removal of the medium, the cultures were washed three times in a phosphate-buffered saline (PBS) buffer. For fixation, 100μl of 4% paraformaldehyde (Beyotime Institute of Biotechnology, Shanghai, China) was added into the wells, incubated for 15 minutes at room temperature (RT), and washed once with 1ml of a PBS buffer. Cell proliferation was evaluated using the EdU Kit in accordance with the manufacturer’s instructions. The results were analyzed by fluorescence microscopy.

### Alkaline phosphatase staining

DMCs were plated in 12-well plates (Corning, Tewksbury MA, USA) at an initial density of 0.5–1×10^4^ cells per well and cultured at 37°C with 5% CO_2_ in an osteogenic induction medium consisting of an alpha-Minimum Essential Medium (α-MEM) supplemented with 5% fetal bovine serum (FBS), 50mg/L of ascorbic acid, 10mmol/L β-glycerophosphate, and 10nmol/L dexamethasone (Sigma-Aldrich, MO, USA). The medium was changed every 3 days. After they were cultured for 7 days, the DMCs were washed in PBS and fixed with 4% paraformaldehyde for 15 minutes. Alkaline phosphatase (ALP) staining was assessed by using an Alkaline Phosphatase Kit (Beyotime Institute of Biotechnology, Shanghai, China). The results were detected by microscopy.

### Von Kossa and alizarin red staining

After osteogenic induction the DMCs were washed with PBS fixed with 4% paraformaldehyde. The mineral deposition was then assessed by staining with a 2% Alizarin red S solution (pH 4.3, Sigma-Aldrich, MO, USA) or a 1% silver nitrate (AgNO_3_) solution on day 14, following a modified version of a previously reported method [19]. The results were detected by microscopy.

### Chondrogenic and adipogenic differentiation of DMCs

DMCs were plated in 12-well plates (Corning, Tewksbury MA, USA) in a chondrogenic differentiation medium (DMEM-High Glucose, Thermo Scientific, Waltham, USA), as well as 1% penicillin and streptomycin (Gibco, Life Technologies, Carlsbad, CA, USA), 10^-7^M dexamethasone (Sigma-Aldrich, MO, USA), 50μg/ml ascorbate- 2-phosphate (Sigma-Aldrich, MO, USA), 0.5mg/ml bovine serum albumin (Sigma-Aldrich, MO, USA), 5μg/ml linoleic acid (Sigma-Aldrich, MO, USA), 10mg/ml insulin, 5.5mg/l transferring, and 5μg/l selenium (ITS) (Sigma-Aldrich, MO, USA). In addition 10ng/ml transforming growth factor-β 3 (TGFβ3) (Sigma-Aldrich, MO, USA) was added. The samples were cultured for 14 days. The DMCs were then washed in PBS and fixed with 4% paraformaldehyde for 15 minutes. Cell staining was assessed by using 0.5% Toluidine Blue (Beyotime Institute of Biotechnology, ShangHai, China). The results were detected by microscopy.

Adipogenesis was induced in the presence of 2mg/ml insulin, 100mM isobutyl methyl xanthine (IBMX), 10mg/ml indomethacin, and 10^−3^M dexamethasone in the basal media of the DMCs. Adipogenic cells were stained after day 14 using Oil Red O (Millipore, Billerica, MA). The results were detected by microscopy.

### Real-time polymerase chain reaction analysis

The total ribonucleic acid (RNA) of two groups was extracted from tissue (mandibles from three-week-old male WT and KO mice) and DMCs using Trizol reagent (Invitrogen Inc, CA). Complementary deoxyribonucleic acid (DNA) was synthesized from RNA by using the PrimeScript RT Reagent Kit (Takara, Dalian, China) and subsequently used in SsoAdvanced^™^ SYBR^®^ Green Supermix real-time polymerase chain reactions (PCR) BioRad Laboratories, Hercules, CA, USA) using a standard protocol. Glyceraldehyde-3-phosphate dehydrogenase (GAPDH) was used as the control. Primer sequences and conditions for real-time polymerase chain reaction are shown in Table 1.

**Table 1:** Primer sequences and conditions for real-time polymerase chain reaction.

Gene	Sequence		Product bp
**SIRT6**	Forward	CTGAGAGACACCATTCTGGACT	188
	Reverse	GGTTGCAGGTTGACAATGACC	
**Gapdh**	Forward	GGAGAGTGTTTCCTCGTCCC	136
	Reverse	ATGAAGGGGTCGTTGATGGC	
**Osx**	Forward	TGAGGAAGAAGCCCATTCAC	198
	Reverse	ACTTCTTCTCCCGGGTGTG	
**PPARΓ**	Forward	CAAGGTGCTCCAGAAGATGA	60
	Reverse	AGTAGCTGCACGTGCTCTGT	
**Bsp**	Forward	ACCCCAAGCACAGACTTTTGA	64
	Reverse	TTTCTGCATCTCCAGCCTTCT	
**OCN**	Forward	AGCAGGAGGGCAATAAGGTA	108
	Reverse	CAAGCAGGGTTAAGCTCACA	
**Bmp2**	Forward	GCTTTTCTCGTTTGTGGA	162
	Reverse	TGGAAGTGGCCCATTTAGAG	
**Ap2**	Forward	GCGTGGAATTCGATGAAATCA	88
	Reverse	CCCGCCATCTAGGGTTATGA	
**ADD1**	Forward	TTTCAGAAGCAGCAGCGAGA	119
	Reverse	TCCTCTTTAGTCCACTTAGTCTTCG	
**Adipoq**	Forward	ATCTGGAGGTGGGAGACCAA	144
	Reverse	GGGCTATGGGTAGTTGCAGT	

The Cellular mitochondrial energy metabolism gene profiles were assessed using the Mouse Mitochondrial Energy Metabolism RT^2^ Profiler^™^ PCR array system (Qiagen, Valencia, CA). The expression values were normalized with 5 housekeeping genes, after which the relative expression of each target gene was calculated by RT^2^ Profiler^™^ PCR array data analysis software. (http://pcrdataanalysis.sabiosciences.com/pcr/arrayanalysis.php).

### Western blot analysis

Two groups of DMCs were washed twice with PBS and scraped in a lysis buffer with a proteinase inhibitor cocktail (Roche R&D Center-China, Shanghai, China). The total protein concentration in the supernatant was measured by a bicinchoninic acid (BCA) Protein Assay Kit (Thermo Scientific, Waltham, USA). 20–40μg were loaded onto a 10% polyacrylamide gel and separated by electrophoresis. The resolved proteins were then transferred to polyvinylidene difluoride (PVDF) membrane blots. After blocking for 1 hour with 5% bull serum albumin (BSA) in tris-buffered saline with 1% Tween-20 (TBST), the PVDF membranes were incubated either with a 1:1000 dilution antibody or an anti-mouse GAPDH antibody (1:1000 dilution, Cell Signaling Technology, MA, USA) at 4°C overnight and washed three times with TBST, followed by a 1 hour incubation period with appropriate horseradish peroxidase (HRP)-conjugated IgG antibodies (1:2000 dilution, Cell Signaling Technology, MA, USA). PVDF membranes were washed with TBST and proteins were visualized using super signal (Pierce Biotechnology, Thermo Scientific, Waltham, USA) enhanced chemiluminescence. For the evaluation of signaling pathway activation, antibodies were applied according to the provided recommendations. The antibodies P-ERK (4370#), ERK (4695#), P-P65 (3033#), P65 (8242#), PP38 (4511#), P38 (9212#), P-JNK (4668#), JNK (9258#) were acquired from Cell Signaling Technology Inc, MA, USA. H3 (ab6147) and H3K9ac (ab4441) were acquired from Abcam company, Cambridge, USA.

### Adenosine Triphosphate (ATP) analysis

Intracellular ATP was measured via the Luciferin /Luciferase method using an ATP Analysis Kit (Beyotime Institute of Biotechnology, Shanghai, China). Briefly, cells were washed with ice-cold PBS and lysed. Luminescence was determined by a luminometer. Cellular protein concentrations were determined using a BCA Protein Assay Kit (Beyotime Institute of Biotechnology, Shanghai, China). ATP contents were calculated according to a standard curve of the supplied ATP standards and normalized using the cellular proteins. Total ATP levels were expressed as μmol/mg protein.

### Statistical analysis

All experiments were repeated at least three times. Results are presented as means ± standard error of the mean (SEM). Dates were analyzed by one-way analysis of variance using SPSS 15.0 software (SPSS Inc, Chicago, IL). A *P* value of <0.05 was considered significant.

## Results

### Deletion of *Sirt6* was associated with retarded development of tooth roots and with retarded eruption

**Fig 1:**
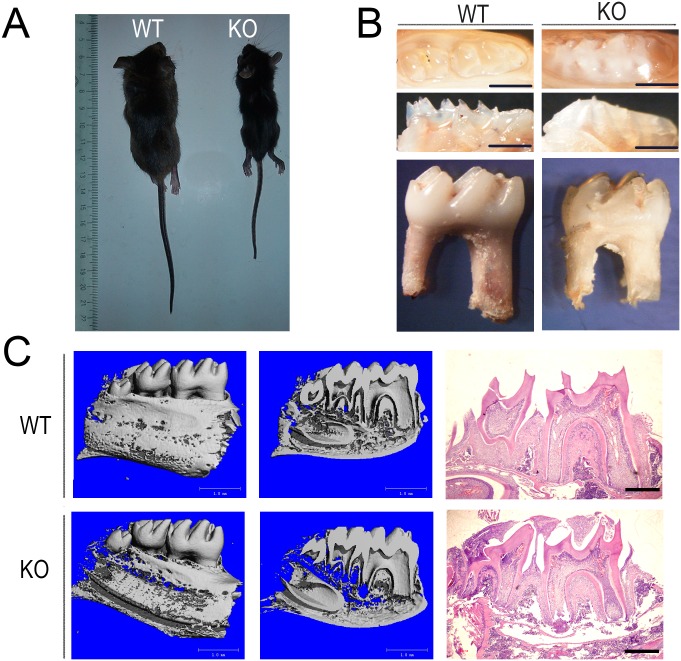
The loss of SIRT6 caused defects in the development of tooth root and tooth eruption. The *Sirt6* KO mice had a smaller body size, thinner subcutaneous fat, and an accelerated mortality at about 3–4 weeks of age. (B) The dental phenotype of both *Sirt6* WT and KO mice showed that both groups had approximately the same tooth contour. Tooth eruption, however, was significantly delayed in the KO mice, and the gingival tissue covering their tooth crown and their tooth root was shorter. (C) Micro-Computed Tomography revealed a reduction in the mandibular bone mineral density of the *Sirt6* KO group. Hematoxylin and eosin (H&E) showed a tendency toward a similar delay in tooth eruption and tooth root development in *Sirt6* KO group. (3W) (n = 6). Bars, as shown at the corner of the images.

**Fig 2:**
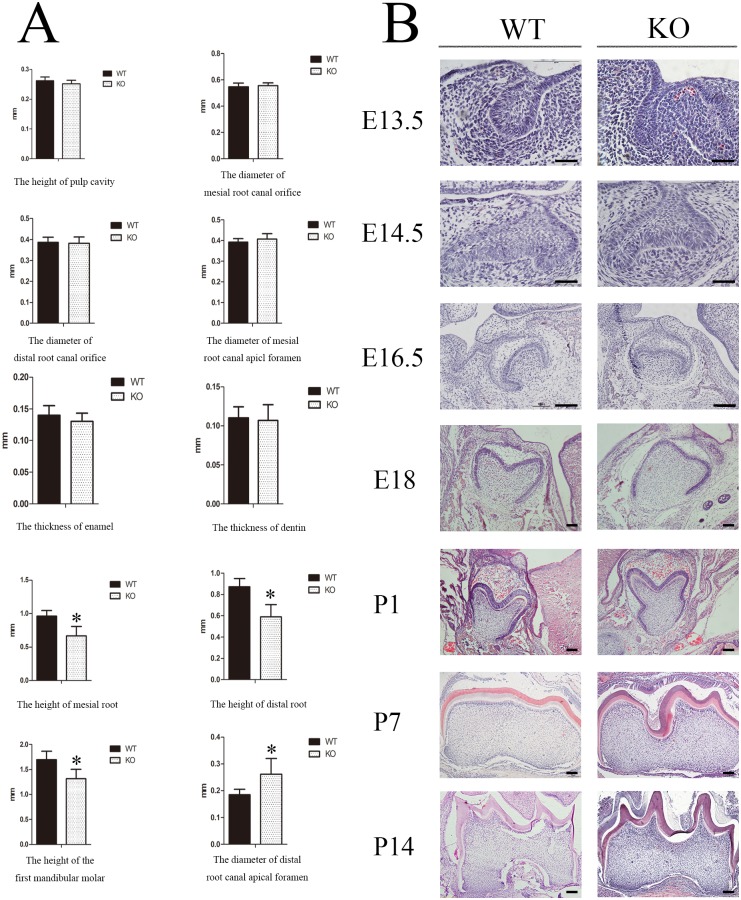
*Sirt6* deletion does not affect the development of the tooth germs. (A) Micro-Computed Tomography testing revealed that the KO group differed from the WT group in several ways. The size of the tooth and mesial and distal roots were reduced in the KO group, and the diameter of the apical foramen of their distal root canal was wider. (B) H&E staining confirmed that there was no significant difference between the two groups with respect to *in utero* tooth germ morphogenesis. However, at or after DPN14, the first mandibular molar of the *Sirt6* KO group suffered an obvious growth retardation and developmental delay when compared to the WT group (E13.5-2W) (n = 6). Bars, as shown at the corner of the images.

Our study revealed that at about 3–4 weeks of age the *Sirt6* homozygous knockout (KO; *Sirt6*^-/-^) mice had a slighter body size, thinner subcutaneous fat, and an accelerated mortality rate. The overall phenotype was similar to what had been previously described [4] (Fig 1A). We first investigated whether *Sirt6* affected tooth development. We used the stereo microscope to observe the morphological structure of the jaw bone and teeth in two groups. When compared with the wild-type (WT; *Sirt6*^+/+^) mice, three-week-old male KO mice showed a roughly similar jaw bone contour. The appearance and length of the incisors in the two groups likewise showed no obvious differences. However, the eruption of molar teeth was significantly delayed in the KO mice. Furthermore there was a delay in the tooth eruption and the tooth root; the roots themselves were also shorter (Fig 1B). H&E staining also revealed a similar tendency (Fig 1C). The result of Micro-Computed Tomography showed that the bone mineral density of the alveolar bone in the KO group was noticeably decreased. Measurement of the physical index of the first mandibular molar by three-dimensional reconstruction images suggested that the height of the tooth, as well as the mesial and distal roots, were reduced in the KO mice. Furthermore the diameter of the apical foramen of the distal root canal was greater than that of the WT mice. There were, however, no significant differences in several other variables, including the thickness of the enamel and dentin, the height of the pulp cavity, the diameter of the mesial/distal root canal orifice, and the diameter of the apical foramen of the mesial root canal (Figs 1C and 2A).

### *Sirt6* deletion does not affect the development of tooth germs

In order to determine the effect of *Sirt6* on tooth development, we carried out histological examination of the development of tooth germs from the bud stage to postnatal day (DPN) 21. The KO group and the WT group did not differ significantly with respect to tooth germ morphogenesis in the bud stage, the cap stage and the bell stage *in utero*. However, at or after DPN14, the formation of the epithelial diaphragm and the tooth root in the first mandibular molar of *Sirt6* KO mice experienced a delay. There was a noticeable retardation in the growth of tooth germs as well as a significant developmental delay. In several other variables, however, the KO and WT mice showed no significant difference. These include cervical-loop structures, cellular morphology and the arrangement of the inner and outer enamel epithelium. (Fig 2B).

### SIRT6 was expressed in mouse odontoblasts and affected the proliferation and differentiation of DMCs

**Fig 3:**
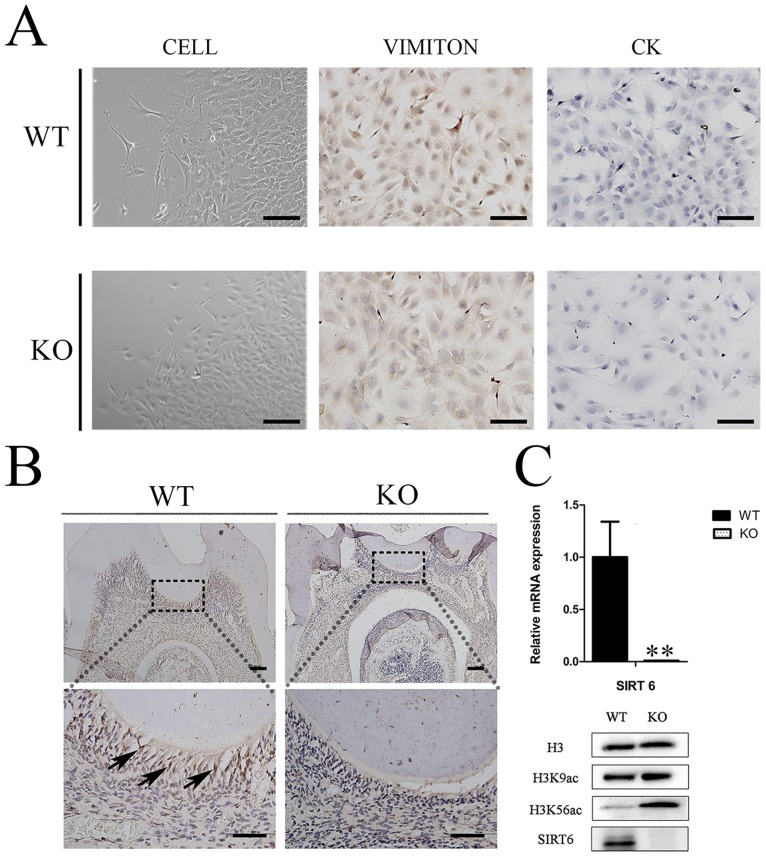
Expression of SIRT6 in mouse dental pulp tissue. (A) The DMCs were either fusiform or triangular in shape; they were positive for vimentin, and negative for cytokeratin. (B) The higher magnification images of Immunohistochemial (IHC) staining showed that SIRT6 was expressed in the nuclei of mature odontoblasts in the WT group. The KO group in contrast showed SIRT6-negative expressions (bars 50μm). Cell nuclei were visualized with hematoxylin. (C) The mRNA and protein levels of *Sirt6* were almost undetectable in the KO mice group. The gene expression level was normalized to GAPDH (n = 6). An assessment ofH3K9ac and H3K56ac in both the WT and KO groups was carried out by western blot analysis. This analysis found that the KO groups experienced a significant increase in these histones. (n≥3) *p<0.05; **p<0.01versus CO. CO = control.

After culture, the cells of the third passage were purified DMCs and were either fusiform or triangular in shape. They were positive for vimentin and negative for cytokeratin, thus confirming their mesenchymal character (Fig 3A). Immunohistochemistry staining demonstrated the SIRT6 that was expressed in the nuclei of mature odontoblasts in WT mice (Fig 3B) and KO mice showed SIRT6-negative expressions. The verification tests showed that the mRNA and protein levels of *Sirt6* were almost undetectable in the KO mice group. Furthermore the SIRT6 substrates, H3K9ac and H3K56ac, were increased in the DMCs of KO mice, but not in those of WT mice (Fig 3C).

**Fig 4:**
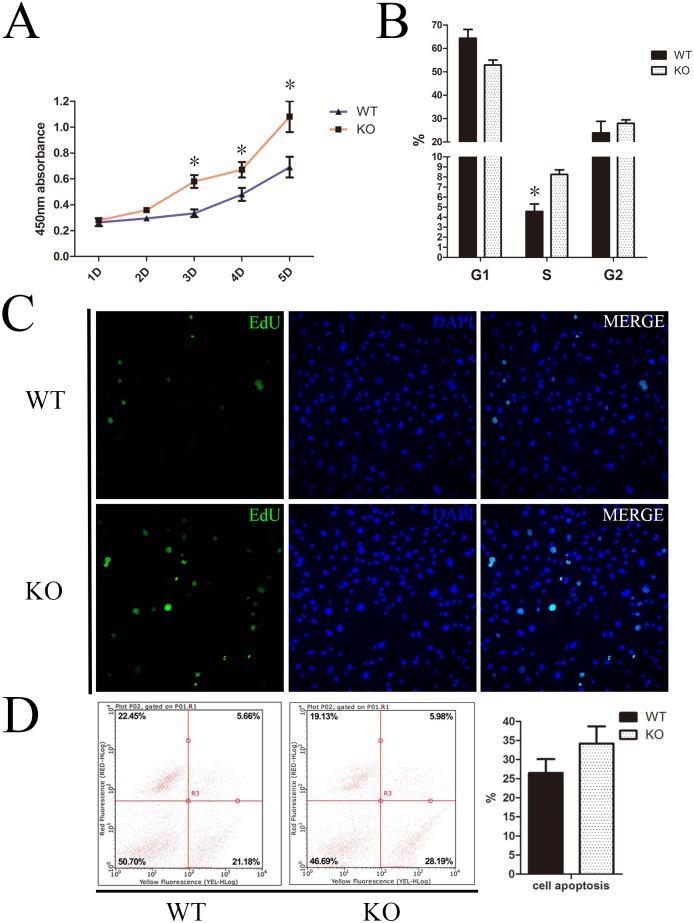
*Sirt6* negatively regulates the proliferation of DMCs. (A) The growth curves of the DMCs indicate that the proliferation of DMCs at day 3–5 was significantly greater in *Sirt6* KO group that than which occurred in the WT group. (B) Flow cytometry analysis revealed that 8.58% of the *Sirt6* KO cells and 4.94% of *Sirt6* WT cells were in the S phase. This difference was statistically significant. (C) An EdU incorporation assay confirmed that the proliferation of DMCs increased significantly in the *Sirt6* KO group at day 4. (D) There was no statistically significant difference in the rate of cell apoptosis between the two groups. *p<0.05 versus CO; CO = control; n = 3; Error bars shown represent the SD.

In order to determine the proliferation ability of DMCs in each of the groups, we cultured DMCs from both the KO and WT group *in vitro*. Growth curves of the DMCs revealed that, when *Sirt6* was knocked out, there was a significant increase in the proliferation of DMCs at day 3–5 in the KO group in contrast with the WT group (Fig 4A). A 5-Ethynyl-2´-deoxyuridine (EdU) incorporation assay confirmed the same result at day 4 (Fig 4C). Flow cytometry analysis revealed that 54.38% of the *Sirt6* KO cells were in the G0/G1 phase, 8.58% in the S phase, and 26.81% in the G2/M phase. By contrast, 66.06% of the *Sirt6* WT cells were in the G0/G1 phase, 4.94% in the S phase, and 20.47% in the G2/M phase (Fig 4B). In addition, the cell apoptosis analysis found that the total rate of apoptosis cells (early apoptosis plus middle-late apoptosis) of the WT group was 26.48%, whereas that of the KO group was 34.17%. This difference, however, was not statistically significant (Fig 4D). In short, these results demonstrated that the proliferation of DMCs in the *Sirt6* KO mice was significantly increased, especially during the proliferative phase in the cell cycle.

**Fig 5:**
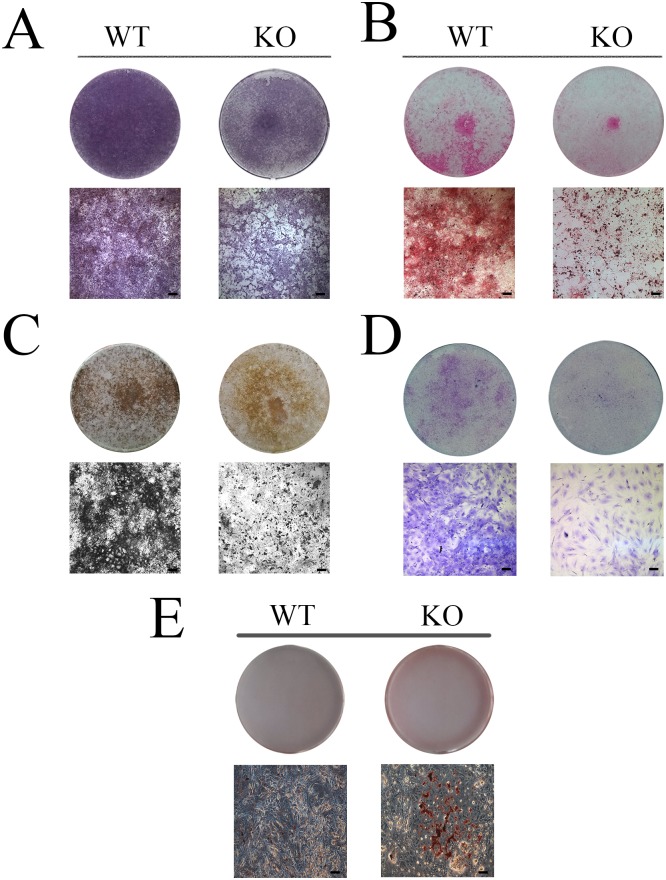
*Sirt6 r*egulates the osteogenic/chondrogenic/ adipogenesis differentiation of DMCs. (A) Alkaline Phosphatase Staining, (B) Alizarin Red Staining, (C) Von Kossa and (D) Toluidine Blue Staining, all of which analyze the differentiation of DMCs, indicate that osteogenic and chondrogenic differentiation is reduced in the *Sirt6* KO group. (E) Oil Red O staining revealed an enhanced ability for adipogenic differentiation in the *Sirt6* KO group.

**Fig 6:**
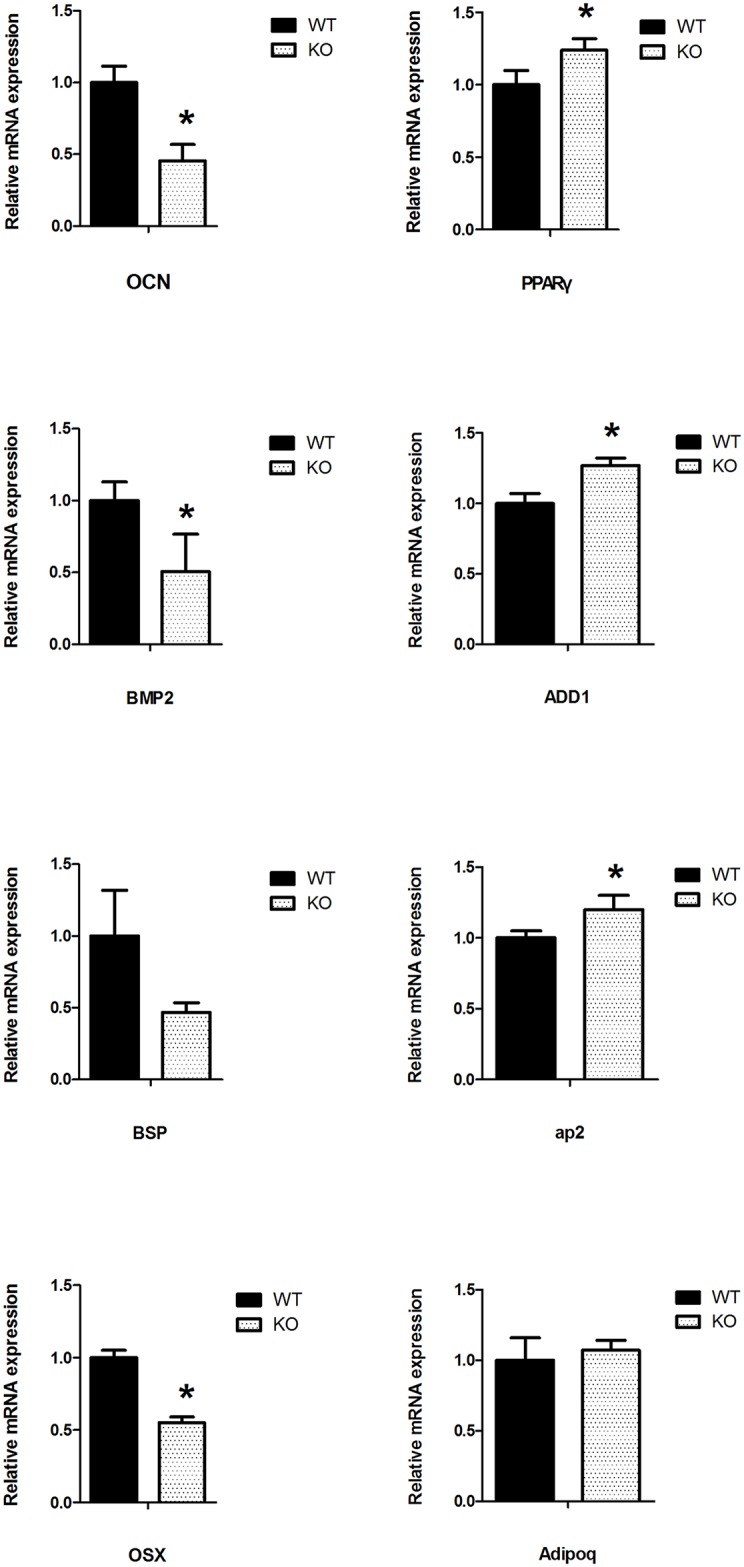
*Sirt6 r*egulates the mRNA levels of the osteogenic/ adipogenic -related transcription factor in the differentiation of DMCs. qPCR revealed that the *Sirt6* KO group experienced a substantial down-regulation in the mRNA levels of the osteogenic-related transcription factor, whereas the levels of the adipogenic-related transcription factor were increased significantly. The gene expression level was normalized to GAPDH (n = 3 to 6), *p<0.05; **p<0.01versus CO; CO = control.

To explore the effect of SIRT6 on the differentiation of DMCs, cells from *Sirt6* WT and KO mice were cultured for 7 and 14 days in a medium conducive to mineralization and to chondrogenic and audiogenic differentiation. ALP staining revealed that the *Sirt6* KO group, but not the *Sirt6* WT group, experienced a reduction in the degree of mineralization after 7 days of culture (Fig 5A). In addition, mineralization assays indicated that, when *Sirt6* is knocked out, mineral nodule formation decreased significantly after 14 days of culture in a mineralization medium (Fig 5B). Furthermore, alizarin red staining revealed that the KO group experienced a significantly lower density of red calcification nodules than the WT groups. Von Kossa and Toluidine Blue staining showed the same result (Fig 5C and 5D). After 14 days of culture, Oil Red O staining displayed an enhanced capacity for adipogenic differentiation in the *Sirt6* KO group (Fig 5E). Furthermore, qPCR analysis revealed that the mRNA levels of OCN, BMP2, BSP and OSX were substantially down-regulated in the *Sirt6* KO group, whereas the adipogenic-related transcription factor PPARγ, ADD1, and Ap2 were increased significantly (Fig 6). These results further supported the hypothesis that SIRT6 affects the differentiation and proliferation of DMCs. It further indicates that *Sirt6* deletion can cause abnormal adipogenesis.

### SIRT6 activated the NF-κB/p65, p38-MAPK and ERK signaling pathways

**Fig 7:**
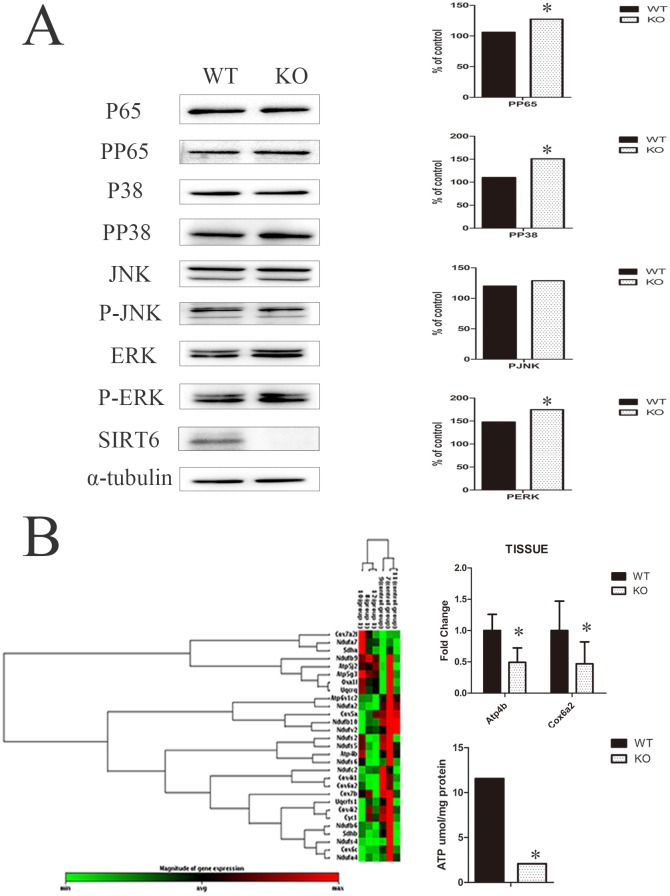
SIRT6 activates the NF-κB/p65, p38-MAPK and ERK signaling pathways and thus affects cellular mitochondrial energy metabolism in DMCs. (A) A western blot analysis of the *Sirt6* KO group showed enhanced activation of the NF-κB/p65, p38-MAPK, and ERK signaling pathways on phosphorylation of those signaling molecules. Quantitative analysis obtained the same relative results. (B) PCR array analysis showed a significant difference between the KO and WT group with respect to the mRNA expression levels of Atp4b and Cox6a2. They were reduced by 51% and 54% in the KO group, whose ATP levels in the DMCs were significantly lower than those of the WT group. The gene expression level was normalized to GAPDH (n = 3). *p<0.05 versus CO. CO = control. GAPDH was used as a control.

To further evaluate the role of *Sirt6* in the *in vitro* proliferation and differentiation of DMCs, we analyzed the activation of associated signaling pathways. The results of the western blot procedure showed an enhanced activation of the NF-κB/p65, p38-MAPK, and ERK signaling pathways. This was demonstrated by phosphorylation of those signaling molecules in the *Sirt6* KO group. But no significant changes were found in the phosphorylation of JNK (Fig 7A). We hypothesize that when SIRT6 is absent, these signaling pathways are activated and exert an impact on the proliferation, differentiation, and apoptosis of cells.

### SIRT6 affects cellular mitochondrial energy metabolism in DMCs

Because SIRT6 can affect cellular energy metabolism, we measured the levels of gene expression associated with the mitochondrial energy metabolism in *Sirt6* WT/KO DMCs (Fig 7B). A scatter plot of normalized data with a minimum 2-fold threshold initially revealed, for the *Sirt6* KO group, a reduction by 51% and 54% of the mRNA expression levels of Atp4b and Cox6a2. (*P*<0.05). The ATP levels of DMCs in the *Sirt6* KO group were thus found to be significantly lower than those of the WT group. These results suggest that SIRT6 may affect the proliferation and differentiation of DMCs by regulating cellular mitochondrial energy metabolism.

## Discussion

The development and eruption of teeth is a complex process that involves multiple determining factors, among the most important of which are the growth of the jaw, the formation of roots, and the traction exerted on periodontal tissue. Our findings reveal that there may be an additional mechanism governing the process of tooth development: the protein-coding *Sirt6* gene. Previous studies had suggested that SIRT6 is active in various cellular pathways, performing functions in transcriptional control, metabolism, and DNA repair. Our work has established that SIRT6 is expressed in mouse odontoblasts and is capable of regulating the proliferation and differentiation of DMCs, SIRT6’s contribution to the process of tooth development appears to occur via interaction with the p38-MAPK, ERK, and NF-κB signal pathways and with the process of mitochondrial energy metabolism.

When the *Sirt6* gene is deleted in KO mice, their intrauterine embryonic development is normal but their growth is retarded after birth [12]. We also found that the development of the mandibular first molar root and the eruption of teeth is delayed in these *Sirt6* KO mice. The morphogenesis of tooth germs was not affected *in utero* by deletion of *Sirt6*, but after birth—specifically at or after DPN14 —a delay occurs in the formation of the epithelial diaphragm and the tooth root of the first mandibular molar. It had already been shown that root development is induced by the growth of Hertwig’s epithelial root sheath (HERS) [20]. During root development, the inner and outer enamel epithelia fuse to form the bilayered HERS, which then induces differentiation of the DF into cementoblasts or osteoblasts [21–24]. As a candidate source for DMCs, DF is also critical for the coordination of tooth eruption [25, 26]. In our research we observed that certain specialized structures in tooth root development, such as the cervical-loop and the inner/outer enamel epithelium, were normal in the *Sirt6* KO group. Therefore, we speculated that *Sirt6* deletion does not affect the formation of such special structures or the cellular morphology in tooth germs. Instead, *Sirt6* deletion exerts an impact on signal pathways and on molecular factors, which in turn result in retardation of tooth development.

SIRT6, located principally in the cellular nucleus, is expressed in the muscles, brain and heart tissue in adult mice [12]. Our experiments, utilizing immunohistochemical staining, found that the SIRT6 protein was localized principally in the nucleus in the pulp tissue of mice and was expressed in the layer of the odontoblast. The fact that its principal expression occurs in the pulp’s odontoblast layer suggests that SIRT6 may be related to dentin formation and mineralization.

Sun et al (2014) found that, after *Sirt6* gene knockout, there was an increase in the cellular proliferation rate of human mesenchymal stem cells and of human dental pulp cells, but a decrease in cell differentiation *in vitro* [27,28]. Additional studies have shown that the proliferation of embryonic fibroblasts (MEFs) was increased significantly and the *Sirt6* knockout mice formed tumor tissue more easily [29]. In addition, through its involvement in the process of histone deacetylation, SIRT6 inhibits glycolysis, triglyceride synthesis, and fat metabolism-related gene expression. It therefore plays a role in the regulation of lipid metabolism [13, 30]. In our study, we used CCK8, EdU, flow cytometry and mineralization assay to measure the proliferation and differentiation of DMCs. The results showed that not only cell proliferation, but also the proportion of cells in DNA replication, were higher in the *Sirt6* KO group than in the WT control group. On the other hand, the capacity of the KO group for osteogenic/chondrogenic differentiation decreased significantly. To verify, we further examined the mRNA expression of related genes in DMCs by qPCR and thus confirmed that cellular differentiation is indeed suppressed. Our result are consistent with previous research indicating a decrease in the bone mass and bone density of knockout mice from whom *Sirt6* had been eliminated[31], SIRT6 most likely contributes to the differentiation of DMCs by modulating osteogenic factors, which may also affect the proliferation of DMCs. At the same time, when sirt6 deletion, the fat metabolism disorder leads to increased triglyceride synthesis and fat, thus enhancing the capacity for adipogenic differentiation.

The signaling pathways associated with mitogen-activated protein kinase (MAPK) play a crucial role in various phases of the cell cycle, including cellular proliferation, differentiation, apoptosis, and transformation. These pathways also mediate biological responses to cellular processes [32–35]. SIRT6 may react with the Rela gene, which is part of the NF-κB complex. In *Sirt6* knockout mice, the NF-κB signaling pathway displayed over-activation [36, 37]. Taking this into account, we hypothesize that SIRT6 and other signal pathways affect the functions of DMCs. In our study, when the *Sirt6* gene was deleted, the NF-κB signaling pathway, as well as the P38-MAPK and ERK in the MAPK signaling pathway, in the DMCs were activated. Therefore, we hypothesize that these signaling pathways interact with SIRT6. This suggests that SIRT6 may play a role in the proliferation and differentiation of DMCs.

As a nuclear protein, SIRT6 has been shown to be responsive to nutrient availability and calorie restriction (CR) [38]. *Sirt6* deficient KO mice have been observed to suffer from severe metabolic defects, such as reduced levels of the serum insulin-like growth factor 1 (IGF-1) and serum glucose. SIRT6 levels were induced following CR in a tissue-specific manner [12,38]. Hypoxia-inducible factor 1-alpha (Hif1α) is a key mediator of the transition that is induced by nutrient and oxygen stress [39,40]. When *Sirt6* was deleted in the KO mice, a down-regulation of Hif1α occurred in cells, accompanied by metabolic shift into glycolysis [41]. In *Sirt6*^-/-^ mice, the expression levels of genes associated with mitochondrial energy metabolism changed in the DMCs. At the same time the mRNA expression levels of Atp4b and Cox6a2 were also significantly reduced.

The Atp4b gene belongs to a family in the protein encoded by the ATP enzyme. This is a proton pump enzyme which catalyzes the hydrolysis of the coupling effect. The protein encoded by the gene Cox6a2 belongs to the group of cytochrome C oxidase enzymes. It is the end point of the mitochondrial respiratory chain enzymes [13,41–43]. Our results suggest that the deletion of the *Sirt6* gene has an impact on the mitochondrial energy metabolism of DMCs which can affect both the formation of ATP and the energy transfer of the mitochondrial respiratory chain enzyme. It is reported that the addition of the dimethyl-α-ketoglutarate (DM-αKG) or the interdiction of the demethylase will change the gene activity of ESCs. Furthermore the epigenetic mechanisms of histone methylation is relevant to osteogenesis and adipogenesis of MSCs [44,45]. We speculate that the deletion of *Sirt6*, because it is a histone deacetylase, may modify the activity and differentiation of DMCs and thus exert an influence on dental development.

In conclusion, we have shown by *in vitro* experimentation that the *Sirt6* gene modulates the function of DMCs. To the best of our knowledge, this is the first study of the relationship between SIRT6 and tooth development. We have given evidence of the role that *Sirt6* plays in tooth root formation and have confirmed the essential role of SIRT6 in DMC differentiation, as well as in the development of tooth roots and in the timing of tooth eruption. Further investigations could therefore be carried out on SIRT6 as a potential mediator in tooth development, and as a potential element for use in dental pulp repair and regeneration.
